# Supplementary material for: Plant photoreceptors and their signaling components compete for COP1 binding via VP peptide motifs
Source: EMBO J. 2019 Jul 15;38(18):e102140. doi: 10.15252/embj.2019102140 (PMC6745501; doi:10.15252/embj.2019102140)
Supplement: Supplementary file 1 — Appendix [file EMBJ-38-e102140-s001.pdf]

Plant photoreceptors compete with COP1 substrates for COP1 binding

Kelvin Lau, Roman Podolec, Richard Chappuis, Roman Ulm, and Michael Hothorn

Appendix Table of Contents

Appendix Figure Legends

Figure S1 - X-ray structures of the COP1 WD40 domains bound to UVR8 and HY5 VP-peptides.

Figure S2 - Interaction analysis of COP1 mutants with UVR8 and HY5 in yeast.

Figure S3 - X-ray crystal structures of COP1 wild-type and COP1^{Lys422Ala} WD40 domains bound to the UVR8 VP-peptide.

Figure S4 - UVR8 is able to bind the COP1 WD40 domain weakly in the absence of UV-B light and the UVR8 core domain is able to bind the COP1 WD40 domain independent of the VP-peptide motif containing C-terminus in response to UV-B by GCI experiments.

Figure S5 - UV-B-activated UVR8$^{ValPro/AlaAla}$ binding to the COP1 WD40 domain is not detectable by ITC experiments and UVR8$^{ValPro/AlaAla}$ does not bind to the COP1 WD40 domain in the absence of UV-B.

Figure S6 - Mutations targeting the COP1 VP-binding site and the UVR8 VP motif both affect binding.

Figure S7 - Coomassie-stained 10% SDS-PAGE gels of purified proteins show high purity.

Figure S8 - Non-light activated CRY2 does not bind COP1.

Table S1 – List of primers and materials

Appendix Figure Legends

Figure S1 - X-ray structures of the COP1 WD40 domains bound to UVR8 and HY5 VP-peptides.

A. The domain organization of the COP1 protein from Arabidopsis. It consists of an N-terminal RING domain followed by a central coiled-coil domain (residues 1-348) and a WD40 domain (residues 349-675).

B. Ribbon diagram of the COP1 WD40 domain (in gray, key residues in blue) bound to the UVR8 VP peptide (in purple).

C. A surface representation of the HY5 peptide binding site of COP1. COP1 is depicted in surface representation, the HY5 peptide is depicted in green in ball-and-stick representation. Selected residues which were mutated in this work (see Fig 1) are highlighted in magenta along with their corresponding accessible surface area.

Figure S2 - Interaction analysis of COP1 mutants with UVR8 and HY5 in yeast.

A, B. Yeast two-hybrid analysis of interactions of COP1 (WT) and COP1 mutants ($COP1^{Lys422Ala}$, $COP1^{Tyr441Ala}$, and $COP1^{Trp467Ala}$) with UVR8 (+/- UV-B), $UVR8^{C44}$ and HY5. Means and SEM for 3 biological repetitions are shown. AD, activation domain; BD, DNA binding domain.

Figure S3 - X-ray crystal structures of COP1 wild-type and $COP1^{Lys422Ala}$ WD40 domains bound to the UVR8 VP-peptide.

A. The crystal structure of the UVR8 VP-peptide depicted in purple in stick representation bound to the COP1 WD40 domain depicted in white also in stick representation. Only selected residues and water molecules in red are shown. The white mesh represents the 2mFo-DFc electron density map contoured around all atoms depicted at a level of 1 σ. The orange mesh represents the polder omit map depicted at a level of 2.5 σ and contoured only around Tyr407 of the UVR8 VP-peptide. Two different conformers of Tyr407 were visible in the electron density and were modeled as shown.

B. The crystal structure of the UVR8 VP-peptide depicted in purple in stick representation bound to the $COP1^{Lys422Ala}$ WD40 domain depicted in white in stick representation. Only selected residues and water molecules in red are shown. The white mesh represents the 2mFo-DFc electron density map contoured around all atoms depicted at a level of 1 σ.

C, D. A surface representation of the UVR8 VP-peptide binding site of (C) wild-type COP1 and (D) $COP1^{Lys422Ala}$. COP1 is depicted in surface representation, the UVR8 peptide is depicted in green in stick representation. Lys422 or Ala422 is highlighted in magenta along with their corresponding accessible surface area.

E, F. Superposition of the X-ray structures of the (E) UVR8 and (F) HY5 VP-peptides bound to the COP1 WD40 domain versus $COP1^{Lys422Ala}$. The UVR8 VP-peptides are depicted in ball-and-stick representation. Selected residues from COP1 are depicted stick representation. The wild-type structure is gray. In the $COP1^{Lys422Ala}$ structure, the peptide is highlighted in purple and the residues in blue.

Figure S4 - UVR8 is able to bind the COP1 WD40 domain weakly in the absence of UV-B light and the UVR8 core domain is able to bind the COP1 WD40 domain independent of the VP-peptide motif containing C-terminus in response to UV-B by GCI experiments.

A – C. Binding kinetics of (A) UVR8 in the absence of UV-B, (B) $UVR8^{12\text{-}381}$ pre-monomerized by UV-B or (C) $UVR8^{12\text{-}415}$ pre-monomerized by UV-B versus the COP1 WD40 domain obtained by GCI. Sensorgrams of UVR8 injected are shown in red, with their respective 1:1 binding model fits in black. The following amounts were typically used: ligand - COP1 (2000 pg/mm^2); analyte – UVR8 and variants (highest concentration 2 μM). k_a = association rate constant, k_d = dissociation rate constant, K_d = dissociation constant.

Figure S5 - UV-B-activated $UVR8^{ValPro/AlaAla}$ binding to the COP1 WD40 domain is not detectable by ITC experiments and $UVR8^{ValPro/AlaAla}$ does not bind to the COP1 WD40 domain in the absence of UV-B.

A. ITC experiment between the COP1 WD40 domain and full-length $UVR8^{ValPro/AlaAla}$ pre-monomerized by UV-B. Integrated heats are shown in solid, cyan squares. The following concentrations were typically used (titrant into cell): $UVR8^{ValPro/AlaAla}$ – COP1 (130 μM in 20 μM).

B. No binding was observed for $UVR8^{ValPro/AlaAla}$ in the absence of UV-B versus the COP1 WD40 domain obtained by GCI experiments. Sensorgrams of $UVR8^{ValPro/AlaAla}$ injected are shown in red. The following amounts were typically used: ligand - COP1 (2000 pg/mm^2); analyte – $UVR8^{ValPro/AlaAla}$ +UV-B (highest concentration 2 μM).

Figure S6 - Mutations targeting the COP1 VP-binding site and the UVR8 VP motif both affect binding.

A, B. Binding kinetics of UVR8 pre-monomerized by UV-B versus the (A) $COP1^{Lys422Ala}$ WD40 domain or (B) $COP1^{Trp467Ala}$ WD40 domain obtained by GCI experiments. Sensorgrams of UVR8 injected are shown in red, with their respective 1:1 binding model fits in black. The following amounts were typically used: ligand - $COP1^{Lys422Ala}$ (2000 pg/mm^2) or - $COP1^{Trp467Ala}$ (4000

pg/mm^2); analyte – UVR8 +UV-B (highest concentration 2 μM). k_a = association rate constant, k_d = dissociation rate constant, K_d = dissociation constant.

C. Binding kinetics of UVR8$^{ValPro/AlaAla}$ pre-monomerized by UV-B versus the COP1^{Trp467Ala} WD40 domain obtained by GCI experiments. Sensorgrams of UVR8 injected are shown in red, with their respective 1:1 binding model fits in black. The following amounts were typically used: ligand - COP1^{Trp467Ala} (2000 pg/mm^2); analyte – UVR8$^{ValPro/AlaAla}$ +UV-B (highest concentration 2 μM). k_a = association rate constant, k_d = dissociation rate constant, K_d = dissociation constant.

Figure S7 - Coomassie-stained 10% SDS-PAGE gels of purified proteins show high purity.

A. Proteins used in Fig 2, Fig EV2 and Appendix Figs 4-6.

B. Proteins used in Figs 1-5, Figs EV2-4 and Appendix Figs S1, S3-6.

C. Proteins used in Fig 2.

D. Proteins used in Fig 3 and Fig EV3.

E. Proteins used in Fig 5 and Appendix Fig S8.

Figure S8 - Non-light activated CRY2 does not bind COP1.

No binding was observed for CRY2 in the absence of supplemental fluorescent light versus the COP1 WD40 domain obtained by GCI experiments. Sensorgrams of CRY2 injected are shown in red. The following amounts were typically used: ligand - COP1 (2000 pg/mm^2); analyte – CRY2 + no additional light (highest concentration 14 μM).

Appendix Figure S1.

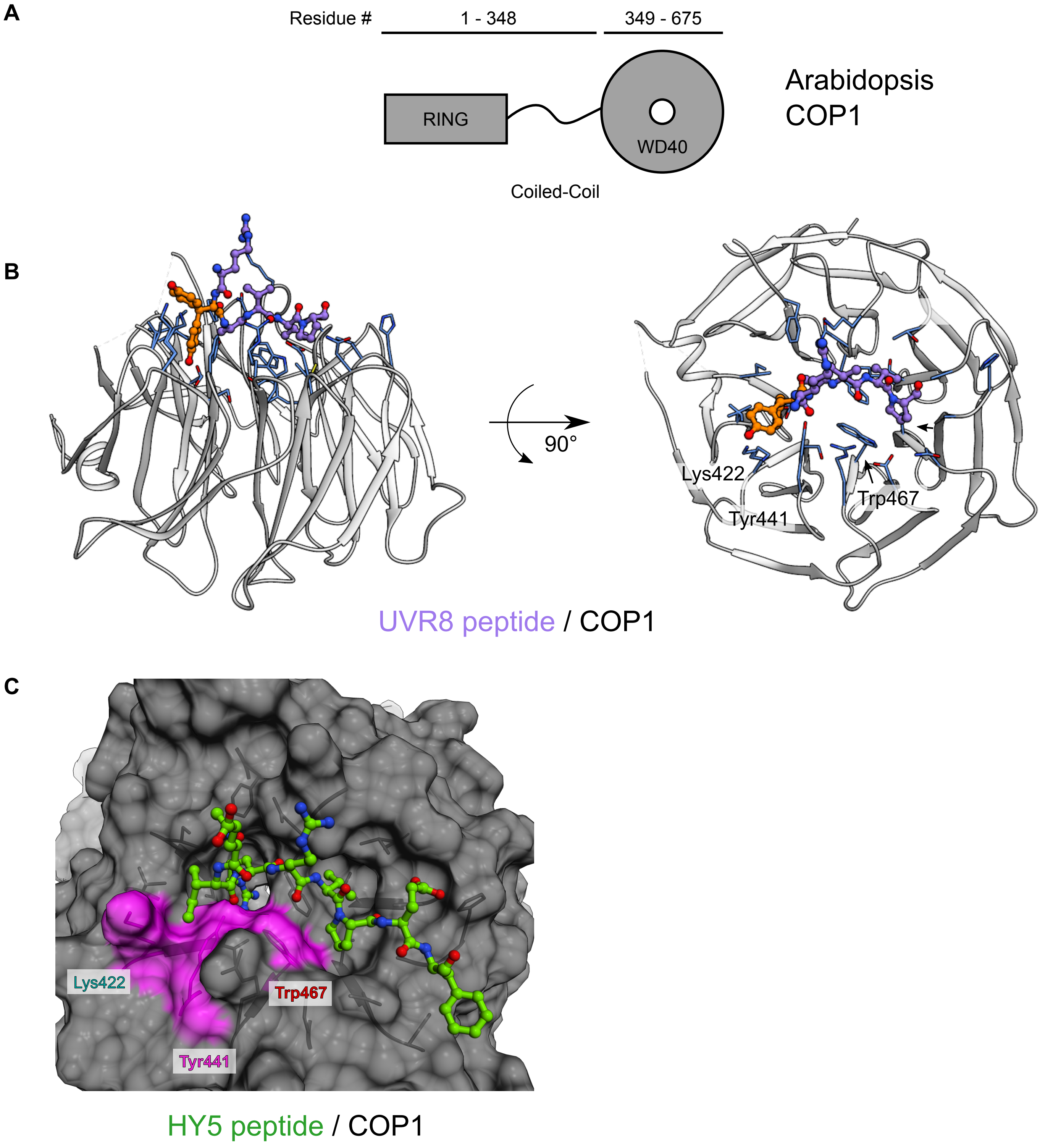

Appendix Figure S2.

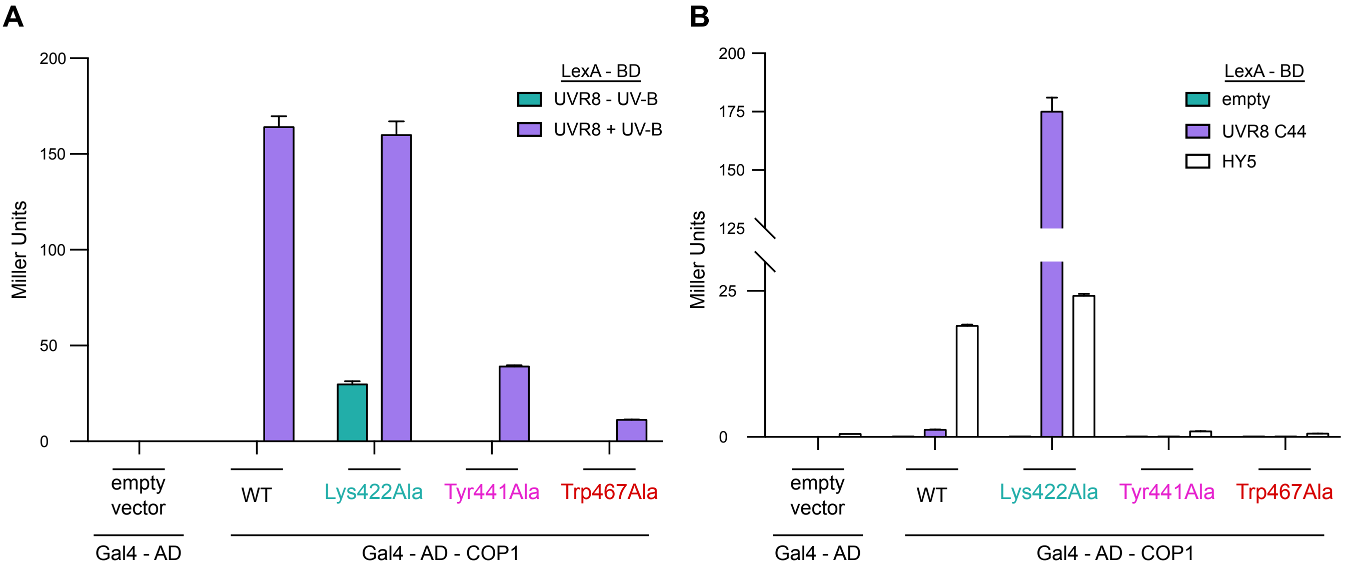

Appendix Figure S3.

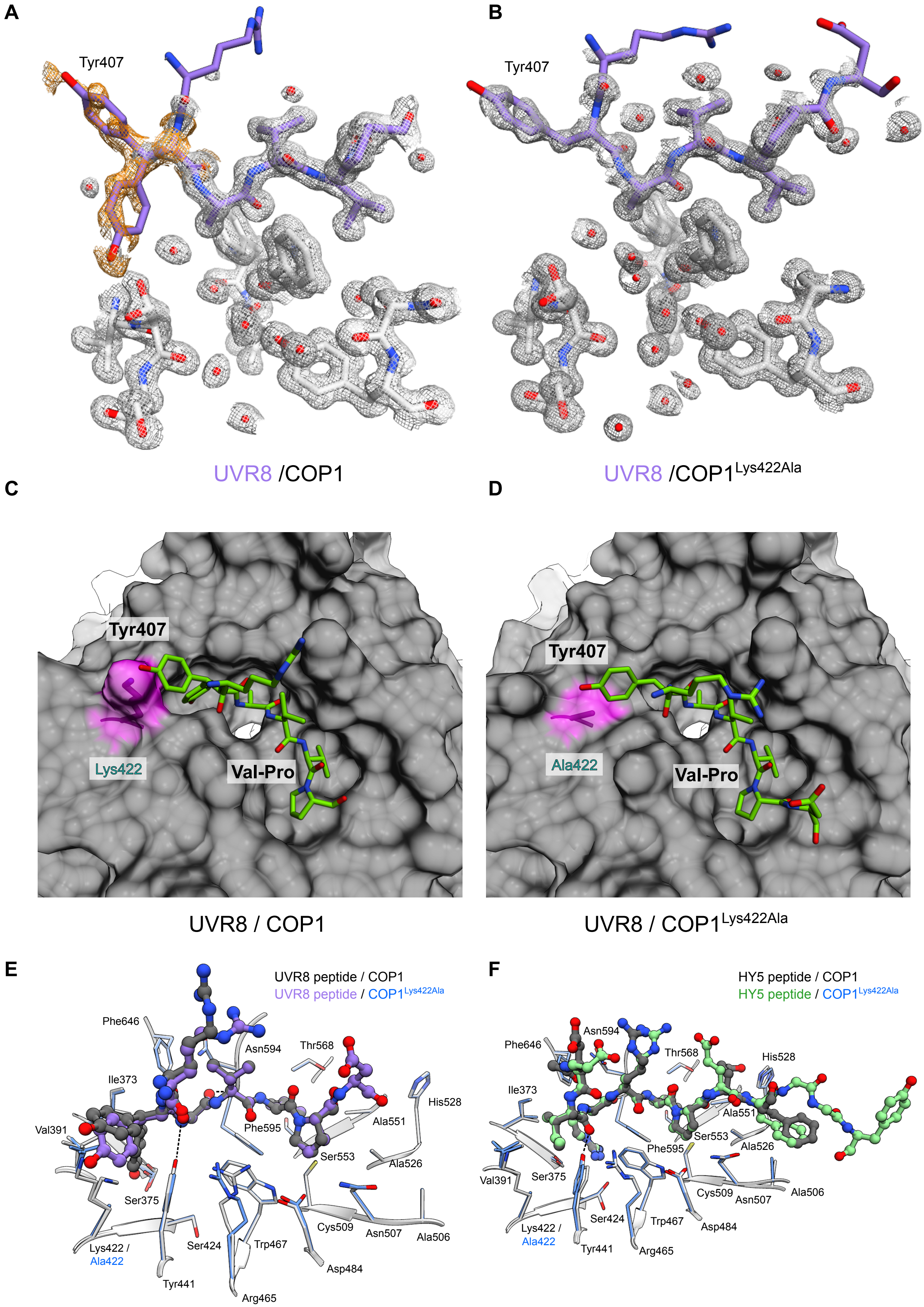

Appendix Figure S4.

A

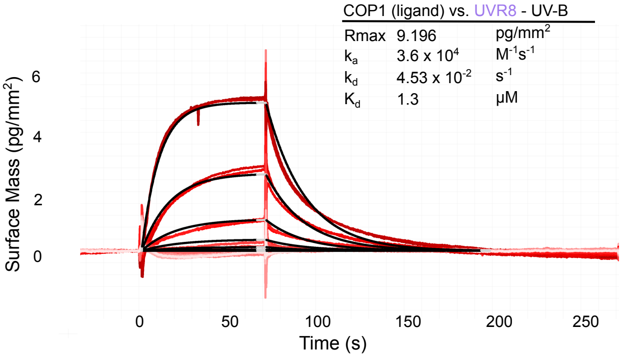

B

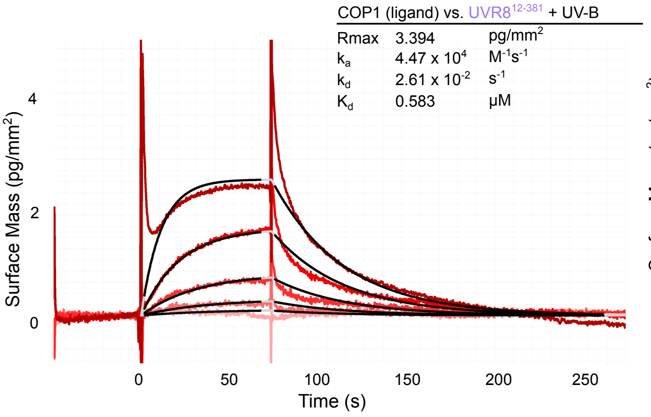

C

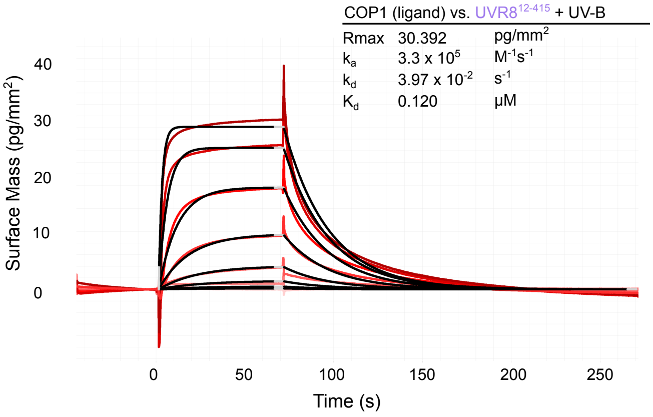

Appendix Figure S5.

A

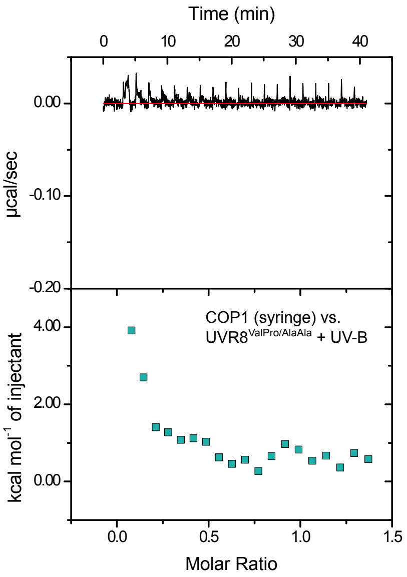

B

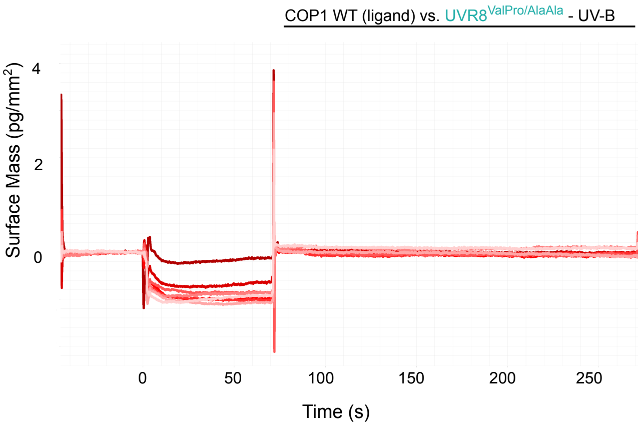

Appendix Figure S6.

A

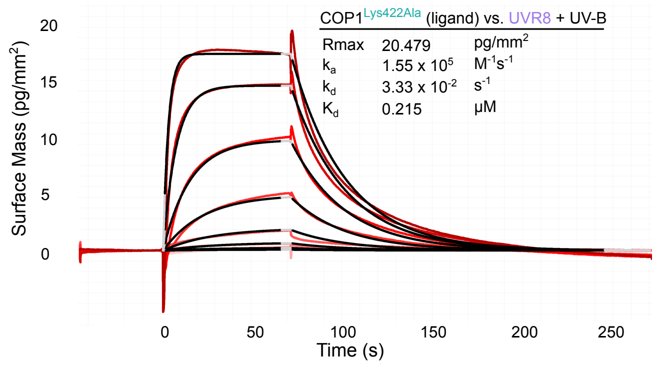

B

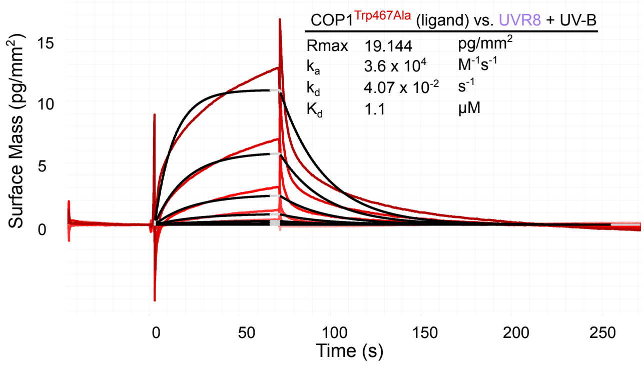

C

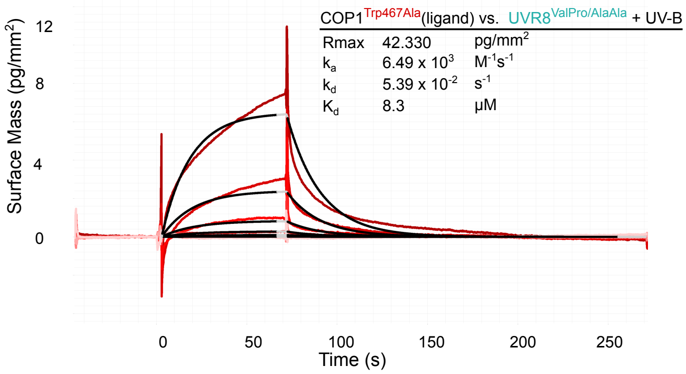

Appendix Figure S7.

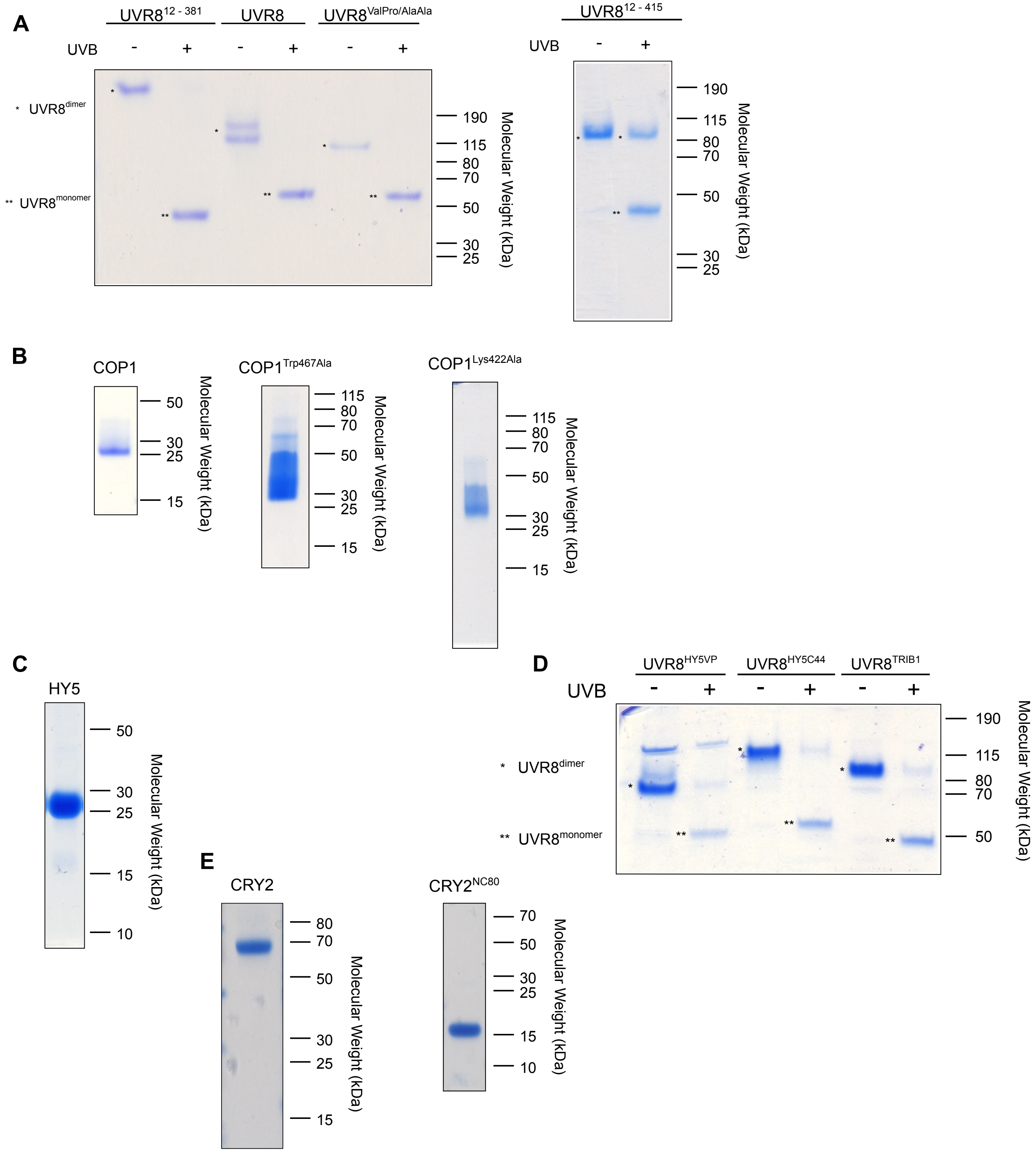

Appendix Figure S8.

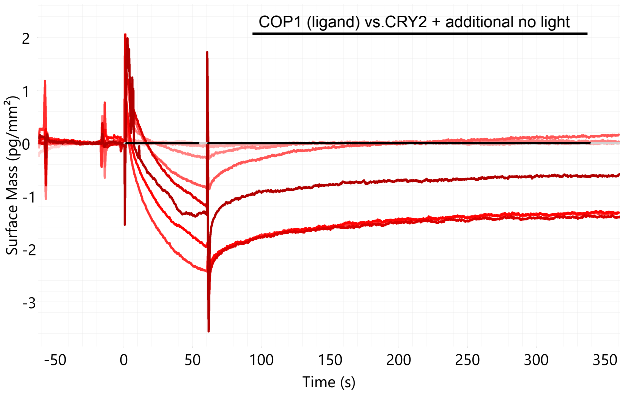

Table S1: List of primers and materials	
**Construction of COP1 yeast/plants vectors**	
COP1_attB_Fw	GGGGACAAGTTTGTACAAAAAAGCAGGCTTCATGGAAG AGATTTCGACGGATCCGG
COP1_attB_Rv	GGGGACCACTTTGTACAAGAAAGCTGGGTCTCACGCAG CGAGTACCAGAACTTTG
COP1_K422A_Fw	GTGGAGATGTCAACTCGGTCTGCACTTAGTTGCTTGAGT TGG
COP1_K422A_Rv	CCAACTCAAGCAACTAAGTGCAGACCGAGTTGACATCTC CAC
COP1_Y441A_Fw	CACATAGCAAGCAGTGATGCTGAAGGAATAGTAACAGT GTGG
COP1_Y441A_Rv	CCACACTGTTACTATTCCTTCAGCATCACTGCTTGCTATG TG
COP1_W467A_Fw	GAGCACGAAAAACGTGCCGCGAGTGTTGACTTTTCACG
COP1_W467A_Rv	CGTGAAAAGTCAACACTCGCGGCACGTTTTTCGTGCTC
**Construction of UVR8 plant vectors**	
UVR8_attB_Fw	GGGGACAAGTTTGTACAAAAAAGCAGGCTCCATGGCG GAGGATATGGCTGCCGAC
UVR8_attB_Rv	GGGGACCACTTTGTACAAGAAAGCTGGGTCTCAAATTC GTACACGCTTGACATCAGTT
UVR8^{HY5C44}_construction_Fw	CTAATATCGATCCATCTTCAATCAAAGAAGGAATTGAAA GCG
UVR8^{HY5C44}_construction_Rv	CGCTTTCAATTCCTTCTTTGATTGAAGATGGATCGATATT AG
UVR8^{HY5C44}_attB_Rv	GGGGACCACTTTGTACAAGAAAGCTGGGTCTCAGACAG TCGCCTGTGTCCG
UVR8^{HY5VP}_construction_Fw	GAGATACGGCGAGTTCCTGAGTTTGGAGGCCTAACGGA TGGTTCAAGC
UVR8^{HY5VP}_construction_Rv	CCTCCAAACTCAGGAACTCGCCGTATCTCCTCTGCAGGC GACACCCAGC
UVR8^{TRIB1}_attB_Rv	GGGGACCACTTTGTACAAGAAAGCTGGGTCTCACTCCT GGTACTCTGGAACAATCTGGTCTGACTCTGCAGGCGAC ACCCAGC
**Construction of yeast three-hybrid pBridge vectors**	
HY5_pBridge_Gal4_Fw	CCTGGATCCAATGCAGGAACAAGCG
HY5_pBridge_Gal4_Rv	GGCGAATTCTCAAAGGCTTGCATC
HYH_pBridge_Gal4_Fw	CTGTATCGCCGGAATTGGATCTCATGTCTCTCCAACGAC CCAATGGG
HYH_pBridge_Gal4_Rv	CATTAACTAGTGAATTCTGGATCTTAGTGATTGTCATCAG TTTTAGG
HFR1-N_pBridge_Gal4_Fw	CTGTATCGCCGGAATTGGATCTCATGTCGAATAATCAAG CTTTCATGG
HFR1-N_pBridge_Gal4_Rv	CATTAACTAGTGAATTCTGGATCTCATTGAAGTTGAAGG TTTTTCATATACTC
UVR8_pBridge_Met25_Fw	GAAAGGTGGCGGCCGGTAGATCCATGGCGGAGGATAT GGCTGCC
UVR8_pBridge_Met25_Rv	CCTACCTAGGCTGCAGAGATCATCAAATTCGTACACGCT TGAC
UVR8$^{1\text{-}396}$_pBridge_Met25_Rv	CCTACCTAGGCTGCAGAGATCATCATGAAGATGGATCG ATATTAG
UVR8^{HY5C44}_pBridge_Met25_Rv	CCTACCTAGGCTGCAGAGATCATCAGACAGTCGCCTGT GTCCG
**Construction of CRISPR/Cas9 vector for CO mutagenesis**	
CO_sgRNA_Fw	ATTGAGACTGGTGGTGGATCAAG
CO_sgRNA_Rv	AAACCTTGATCCACCACCAGTCT
**qRT-PCR**	
HY5_Fw	AAAGAAGGAATTGAAAGCGATGA
HY5_Rv	AAGTTTCTTTTCCGACAGCTTCTC
RUP2_Fw	TGAATTCGATCCCACTGATAACA

RUP2_Rv	AGGGAGGCCGTAAAAACGA
CHS_Fw	CGTGTTGAGCGAGTATGGAAAC
CHS_Rv	TGACTTCCTCCTCATCTCGTCTAGT
ELIP2_Fw	GTGAGTACGAAGTTTGGAGATTTGC
ELIP2_Rv	TTGCTAGTCTCCCGTTGATCCT
UVR8_Fw	GGAGAGGATGGACAGTTAGGTCAT
UVR8_Rv	CACAGGTAACGGAAACAATTTGG
COP1_Fw	AGCCAACCACTTCATGTCTTCA
COP1_Rv	ATCCCATAAGCGTAGTGTGCTATCT
18S_Fw	TGGAGGGCAAGTCTGGTGCC
18S_Rv	CGGCCGACCCATCCCAAGG
**Protein constructs for expression**	
NcoI - COP1-Sf9-N349	ATATTACCATGGCCACTTTCACCCGCTACTCC
NotI-COP1-Sf9-C675	ATATTGCGGCCGCTTAAGCAGCCAGCACCAGCACCTTG A
AtCOP1 K422A for	TCCGCGCTGTCCTGCCTGTCTTGGAACAAGCA
AtCOP1 K422A rev	ACAGCGCGGAACGGGTGGACATCTCGAC
AtCOP1 W467A F	CGCTGCGAGCGTGGACTTCTCCCGTACCG
AtCOP1 W467A R	GCTCGCAGCGCGCTTCTCGTGTTCCTCGTATTCC
AtCOP1 Y441A F	CGACGCCGAGGGTATCGTCACCGTGTGGG
AtCOP1 Y441A R	CTCGGCGTCGGAAGAGGCGATGTGGTTCTTT
NcoI-AtUVR8_WT_Sf9-N12	ATATATCCATGGCTCCCCCTAGGAAGGT
NotI-AtUVR8_WT_Sf9-C381	ATATAGCGGCCGCTTAACCGTCCACAGACAGGG
NotI-AtUVR8_WT_Sf9_C415	TATATGCGGCCGCTTATCCAGTCTCGTCGGGCAC
234_AtUVR8_Gibson_F	CGAGAACCTGTACTTCCAAGGTGCCATGGCGGAGGATA TGGCTGC
233_AtUVR8_HY5_C44_Gibson_R	TTCGATCCTACTCGAGTGCGGCCGCTCAGACAGTCGCC TGTGTCCGC
235_AtUVR8_HY5_VP_Gibson_R	CCTACTCGAGTGCGGCCGCTCAAATTCGTACACGCTTGA CATCAGTTTG
236_AtUVR8_TRIB1_VP_Gibson_R	ATCCTACTCGAGTGCGGCCGCTCACTCCTGGTACTCTGG AACAATCTG
223_AtUVR8_Sf9_VP1_AA_F_new	GTGGCGGCCGACGAGACTGGACTGACCGACGGTTCCT CCAAGGGCA
224_AtUVR8_Sf9_VP1_AA_R_new	CTCGTCGGCCGCCACAGCGTAACGCTCAGCAGGGGAC ACCCAGGA
230_AtCRY2_pBB3_Gibson_F	GAGAACCTGTACTTCCAAGGTGCCATGAAGATGGACAA AAAGACTATAG
229_AtCRY2_pBB3_Gibson_R	TCGATCCTACTCGAGTGCGGCCGCTCATTTGCAACCATT TTTTCCCAAA

**REAGENT**	SOURCE	CODE
**Antibodies**		
anti-actin (mouse) antibody	Sigma	A0480
anti-GFP (mouse) antibody	Clontech	632381
anti-CRY2$^{(588-602)}$ (rabbit) antibody	Eurogentec, this study	N/A
anti-UVR8$^{(1-15)}$ (rabbit) antibody	Yin et al., 2015	N/A
anti-UVR8$^{(410-424)}$ antibody	Heijde and Ulm, 2013	N/A
anti-UVR8$^{(426–440)}$ (rabbit) antibody	Favory et al., 2009	N/A
polyclonal anti-mouse (goat) antibody, HRP-conjugated	Dako	P0447
polyclonal anti-rabbit (swine) antibody, HRP-conjugated	Dako	P0399
**Peptides and Recombinant Proteins**		
Peptide: UVR8 (Ac-RYAVVPDE-NH2) aa: 406-413	Peptide Speciality Labs	N/A

Peptide: HY5 (Ac-EIRRVPEFGGY-NH2) aa: 39-48 Note: a Tyrosine residue was attached for concentration measurements	Peptide Speciality Labs	N/A
Peptide: STO (Ac-EHFIVPDLY-NH2) aa: 240-247 Note: a Tyrosine residue was attached for concentration measurements	Peptide Speciality Labs	N/A
Peptide: HFR1 (Ac-YLQIVPEIHK-NH2) aa: 57-64	Peptide Speciality Labs	N/A
Peptide: HYH (Ac-ELLMVPDMY-NH2) aa: 27-34 Note: a Tyrosine residue was attached for concentration measurements	Peptide Speciality Labs	N/A
Peptide: CRY1 (Ac-EDQMVPSITY-NH2) aa: 544-551 Note: a Tyrosine residue was attached for concentration measurements	Peptide Speciality Labs	N/A
Peptide: CRY2 (Ac-NDQQVPSAVY-NH2) aa: 527-535 Note: a Tyrosine residue was attached for concentration measurements	Peptide Speciality Labs	N/A
Peptide: COL3 (Ac-GFGVVPSFY-NH2) aa: 287-294 Note: a Tyrosine residue was attached for concentration measurements	Peptide Speciality Labs	N/A
Peptide: CO (Ac-GYGIVPSFY-NH2) aa: 366-373 Note: a Tyrosine residue was attached for concentration measurements	Peptide Speciality Labs	N/A
TRIB1 (Ac-SDQIVPEY-NH2) aa: 354-361	Peptide Speciality Labs	N/A
Recombinant protein: arabidopsis COP1 WD40 (aa 349-675)	This study	N/A
Recombinant protein: arabidopsis COP1 WD40 Lys422Ala (aa 349-675)	This study	N/A
Recombinant protein: arabidopsis COP1 WD40 Tyr441Ala (aa 349-675)	This study	N/A
Recombinant protein: arabidopsis COP1 WD40 Trp467Ala (aa 349-675)	This study	N/A
Recombinant protein: arabidopsis UVR8 full-length (aa 1-440)	This study	N/A
Recombinant protein: arabidopsis UVR8 core domain (aa 12-381)	This study	N/A
Recombinant protein: arabidopsis UVR8 with VP (aa 12-415)	This study	N/A
Recombinant protein: arabidopsis UVR8 full-length Val410Pro411/AlaAla (aa 1-440)	This study	N/A
Recombinant protein: arabidopsis UVR8 chimera HY5VP (see Figure 3)	This study	N/A

Recombinant protein: arabidopsis UVR8 chimera HY5C44 (see Figure 3)	This study	N/A
Recombinant protein: arabidopsis UVR8 chimera TRIB1 (see Figure 3)	This study	N/A
Recombinant protein: arabidopsis HY5 full-length (see Figure 3)	This study	N/A
Recombinant protein: arabidopsis CRY2 full-length (aa 1 - 612)	This study	N/A
Recombinant protein: arabidopsis CRY2 NC80 (aa 486 - 565)	This study	N/A
**Deposited Data**		
$HY5^{39-48}$ – $COP1^{349-675}$	This study	PDB: **6QTO**
$UVR8^{406-413}$ – $COP1^{349-675}$	This study	PDB: **6QTQ**
$HY5^{39-48}$ – $COP1^{349-675,Lys422Ala}$	This study	PDB: **6QTR**
$UVR8^{406-413}$ – $COP1^{349-675,Lys422Ala}$	This study	PDB: **6QTS**
HYH^{27-34} – $COP1^{349-675}$	This study	PDB: **6QTT**
$STO^{240-247}$ – $COP1^{349-675}$	This study	PDB: **6QTU**
$HFR1^{57-64}$ – $COP1^{349-675}$	This study	PDB: **6QTV**
$CRY1^{544-552}$ – $COP1^{349-675}$	This study	PDB: **6QTW**
$COL3^{287-294}$ – $COP1^{349-67}$	This study	PDB: **6QTX**
**Organisms/Strains**		
Spodoptera frugiperda (Sf9)	Thermofisher	11496015
Yeast: L40	Vojtek and Hollenberg, 1995	N/A
Yeast: Y187	Harper et al, 1993	N/A
Yeast: Y190	Harper et al, 1993	N/A
Plant: *co-11* (Ws)	This study	N/A
Plant: *cop1-4* (Ws)	Oravecz et al., 2006	N/A
Plant: *cop1-5* (Ws)	McNellis et al., 1994	N/A
Plant: *cop1-5/* Pro_{35S}*:YFP-COP1*	This study	N/A
Plant: *cop1-5/* Pro_{35S}*:YFP-*$COP1^{Lys422Ala}$	This study	N/A
Plant: *cop1-5/* Pro_{35S}*:YFP-*$COP1^{Tyr441Ala}$	This study	N/A
Plant: *cop1-5/* Pro_{35S}*:YFP-*$COP1^{Trp467Ala}$	This study	N/A
Plant: *cry2-1* (Col)	Guo et al., 1998	N/A
Plant: *uvr8-7* (Ws)	Favory et al., 2009	N/A
Plant: *uvr8-7*/Pro_{35S}*:*$UVR8^{HY5C44}$	This study	N/A
Plant: *uvr8-7*/Pro_{35S}*:*$UVR8^{HY5VP}$	This study	N/A
Plant: *uvr8-7*/Pro_{35S}*:*$UVR8^{TRIB1}$	This study	N/A
Plant: Ws	Arabidopsis Biological Resource Center (ABRC)	N/A
Plant: Col-0	Arabidopsis Biological Resource Center (ABRC)	N/A
**Oligonucleotides**		
See Table S1	See Table S1	N/A
**Recombinant DNA**		
COP1 and mutant variants (protein expression)	Geneart	N/A
UVR8 and variants (protein expression)	Geneart	N/A

HY5 (protein expression)	Geneart	N/A
CRY2 and variants (protein expression)	Twist Bioscience	N/A
pGADT7-GW (yeast)	Marrocco et al., 2006	N/A
pGADT7-COP1 (yeast)	Yin et al., 2015	N/A
pGADT7-COP1 mutant variants (yeast)	This study	N/A
pBTM116-D9-GW (yeast)	Stelzl et al., 2005	N/A
pBTM116-HY5 (yeast)	Binkert et al., 2016	N/A
pBTM116-UVR8 (yeast)	Yin et al., 2015	N/A
pBTM116-UVR8^{C44} (yeast)	Yin et al., 2015	N/A
pBridge-HY5-UVR8 (yeast)	This study	N/A
pBridge-HY5-UVR8$^{ValPro/AlaAla}$ (yeast)	This study	N/A
pBridge-HY5-UVR8$^{1\text{-}396}$ (yeast)	This study	N/A
pBridge-HY5-UVR8^{HY5C44} (yeast)	This study	N/A
pBridge-HYH-UVR8 (yeast)	This study	N/A
pBridge-HFR1N-UVR8 (yeast)	This study	N/A
pB7WGY2-COP1 and mutant variants (plant)	This study	N/A
pB2GW7-UVR8 chimeras (yeast/protein expression)	This study	N/A
pHEE401E-COsgRNA (plant)	This study	N/A
Modified pFastBac vector for Bacmid production	This study	N/A
