# Supplementary material for: Plant photoreceptors and their signaling components compete for COP1 binding via VP peptide motifs
Source: EMBO J. 2019 Jul 15;38(18):e102140. doi: 10.15252/embj.2019102140 (PMC6745501; doi:10.15252/embj.2019102140)
Supplement: Supplementary file 2 — Expanded View Figures PDF [file EMBJ-38-e102140-s002.pdf]

Expanded View Figures

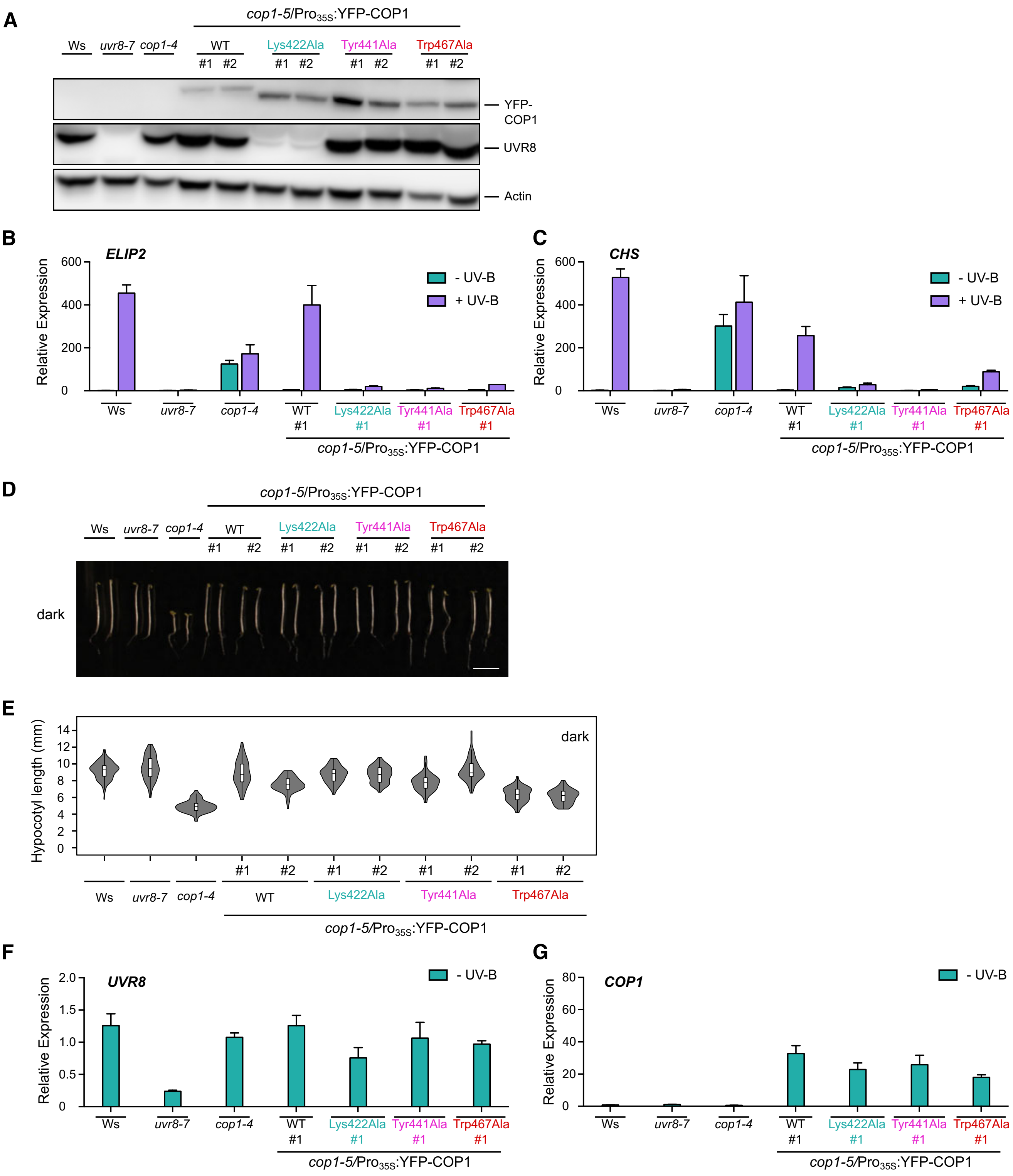

Figure EV1.

Figure EV1. Characterization of COP1 mutant lines.

A Immunoblot analysis of YFP-COP1, UVR8, and actin (loading control) protein levels in lines shown in Fig 1F. Seedlings were grown for 4 days under white light.

B, C Quantitative real-time PCR analysis of (B) *ELIP2* and (C) *CHS* expression. Four-day-old seedlings grown in white light were exposed to narrowband UV-B for 2 h (+ UV-B) or not (− UV-B). Error bars represent SEM of three biological replicates.

D, E Images of representative individuals (D) and quantification of hypocotyl lengths (E) of 4-day-old seedlings grown in darkness. The scale bar represents 5 mm. Violin and box plots are shown for $n > 60$ seedlings; upper and lower hinges correspond to the first and third quartiles; the horizontal line in the interior of the box indicates the median.

F, G Quantitative real-time PCR analysis of (F) *UVR8* and (G) *COP1* expression in wild type (Ws), *uvr8-7*, *cop1-4* and *cop1-5/Pro*$_{35S}$*:YFP-COP1* (WT), *cop1-5/Pro*$_{35S}$*:YFP-COP1*^{Lys422Ala}, *cop1-5/Pro*$_{35S}$*:YFP-COP1*^{Tyr441Ala}, and *cop1-5/Pro*$_{35S}$*:YFP-COP1*^{Trp467Ala} seedlings grown for 4 days under white light. Error bars represent SEM of 3 biological replicates.

Data Information: In (A–G), lines used: wild type (Ws), *uvr8-7*, *cop1-4, cop1-5*/Pro$_{35S}$:*YFP-COP1* (WT), *cop1-5*/Pro$_{35S}$:*YFP-COP1*^{Lys422Ala}, *cop1-5*/Pro$_{35S}$:*YFP-COP1*^{Tyr441Ala}, and *cop1-5*/Pro$_{35S}$:*YFP-COP1*^{Trp467Ala} seedlings. #1 and #2: independent transgenic lines.

Source data are available online for this figure.

 © 2019 The Authors

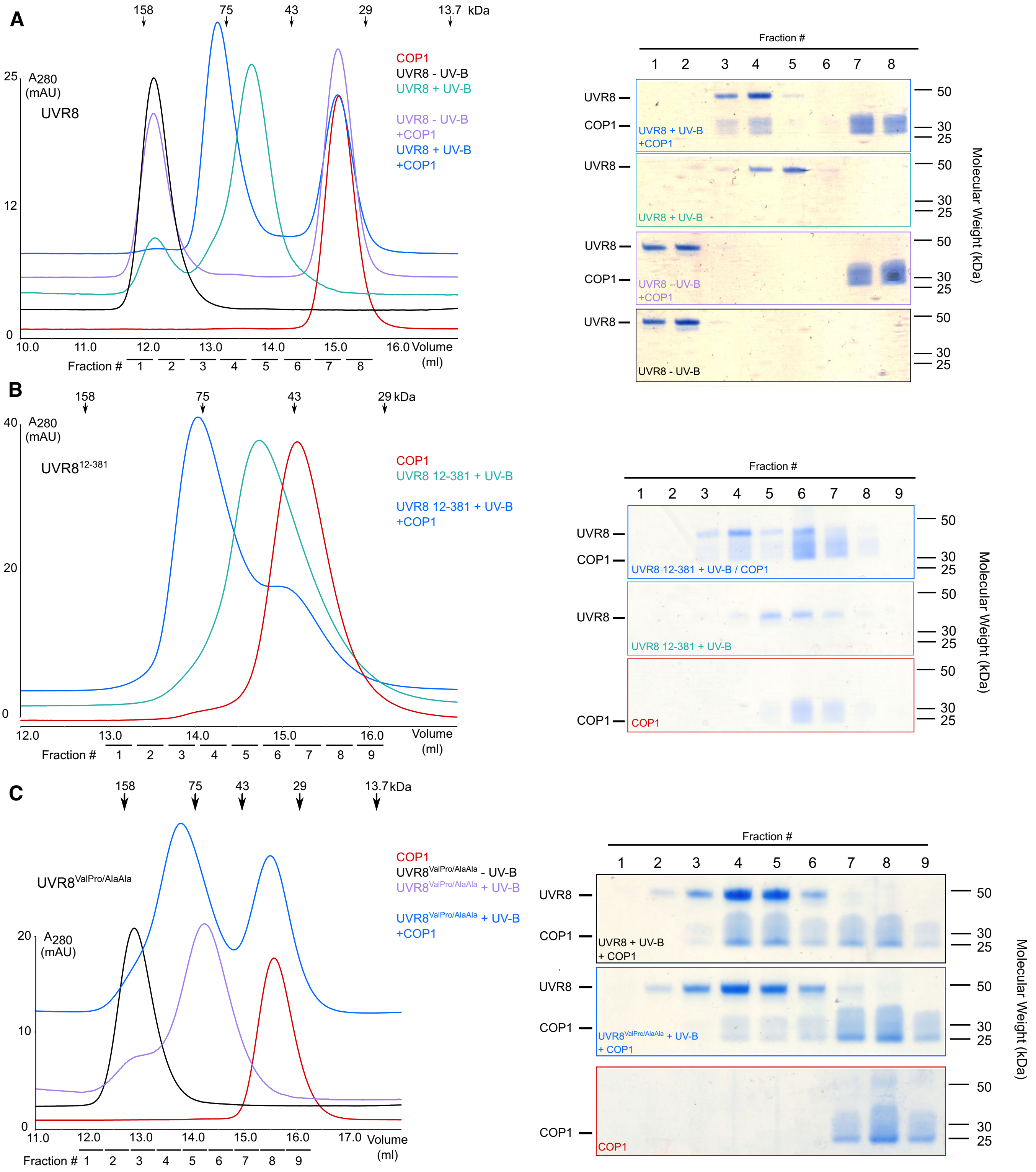
A
158
75
43
29
13.7 kDa
25
A280
(mAU)
UVR8
12
0
10.0
11.0
12.0
13.0
14.0
15.0
16.0 Volume
(ml)
Fraction # 1 2 3 4 5 6 7 8
COP1
UVR8 - UV-B
UVR8 + UV-B
UVR8 - UV-B
+COP1
UVR8 + UV-B
+COP1
Fraction #
1 2 3 4 5 6 7 8
UVR8
COP1
UVR8 + UV-B
+COP1
UVR8
UVR8 + UV-B
UVR8
COP1
UVR8 - UV-B
+COP1
UVR8
UVR8 - UV-B
50
30
25
Molecular Weight (kDa)
B
158
75
43
29 kDa
40
A280
(mAU)
UVR8 12-381
20
0
12.0
13.0
14.0
15.0
16.0
Volume
(ml)
Fraction # 1 2 3 4 5 6 7 8 9
COP1
UVR8 12-381 + UV-B
UVR8 12-381 + UV-B
+COP1
Fraction #
1 2 3 4 5 6 7 8 9
UVR8
COP1
UVR8 12-381 + UV-B / COP1
UVR8
UVR8 12-381 + UV-B
COP1
COP1
C
158
75
43
29
13.7 kDa
UVR8 ValPro/AlaAla
20
A280
(mAU)
10
0
11.0
12.0
13.0
14.0
15.0
16.0
17.0
Volume
(ml)
Fraction # 1 2 3 4 5 6 7 8 9
COP1
UVR8 ValPro/AlaAla - UV-B
UVR8 ValPro/AlaAla + UV-B
UVR8 ValPro/AlaAla + UV-B
+COP1
Fraction #
1 2 3 4 5 6 7 8 9
UVR8
COP1
UVR8 + UV-B
+ COP1
UVR8
COP1
UVR8 ValPro/AlaAla + UV-B
+ COP1
COP1
COP1

Figure EV2.

Figure EV2. Only UV-B-activated UVR8 and mutants are able to bind COP1 in size-exclusion chromatography binding assays.

A Coomassie-stained SDS–PAGE gels from a size-exclusion chromatography binding assay between the COP1 WD40 domain and UVR8 in the presence and absence of UV-B. Purified monomeric UVR8 ~ 50 kDa. Four µM of each protein or a mix of proteins was loaded on to a Superdex 200 Increase 10/300 GL column. Indicated fractions were taken each of the size-exclusion chromatography runs and separated on a 10% SDS–PAGE gel.

B Coomassie-stained SDS–PAGE gels from a size-exclusion chromatography binding assay between the COP1 WD40 domain and UVR8$^{12–381}$ in the presence and absence of UV-B. Purified monomeric UVR8$^{12–381}$ ~ 40 kDa. Four µM of each protein or a mix of proteins was loaded on to a Superdex 200 Increase 10/300 GL column. Indicated fractions were taken each of the size-exclusion chromatography runs and separated on a 10% SDS–PAGE gel.

C Coomassie-stained SDS–PAGE gels from a size-exclusion chromatography binding assay between COP1 and UVR8$^{ValPro/AlaAla}$ pre-monomerized by UV-B. Purified monomeric UVR8$^{ValPro/AlaAla}$ ~ 50 kDa, COP1 WD40 a smear ~ 25–40 kDa. Four µM of each protein was loaded independently or mixed together. Indicated fractions were taken each of the size-exclusion chromatography runs and separated on a 10% SDS–PAGE gel.

Source data are available online for this figure.

 © 2019 The Authors

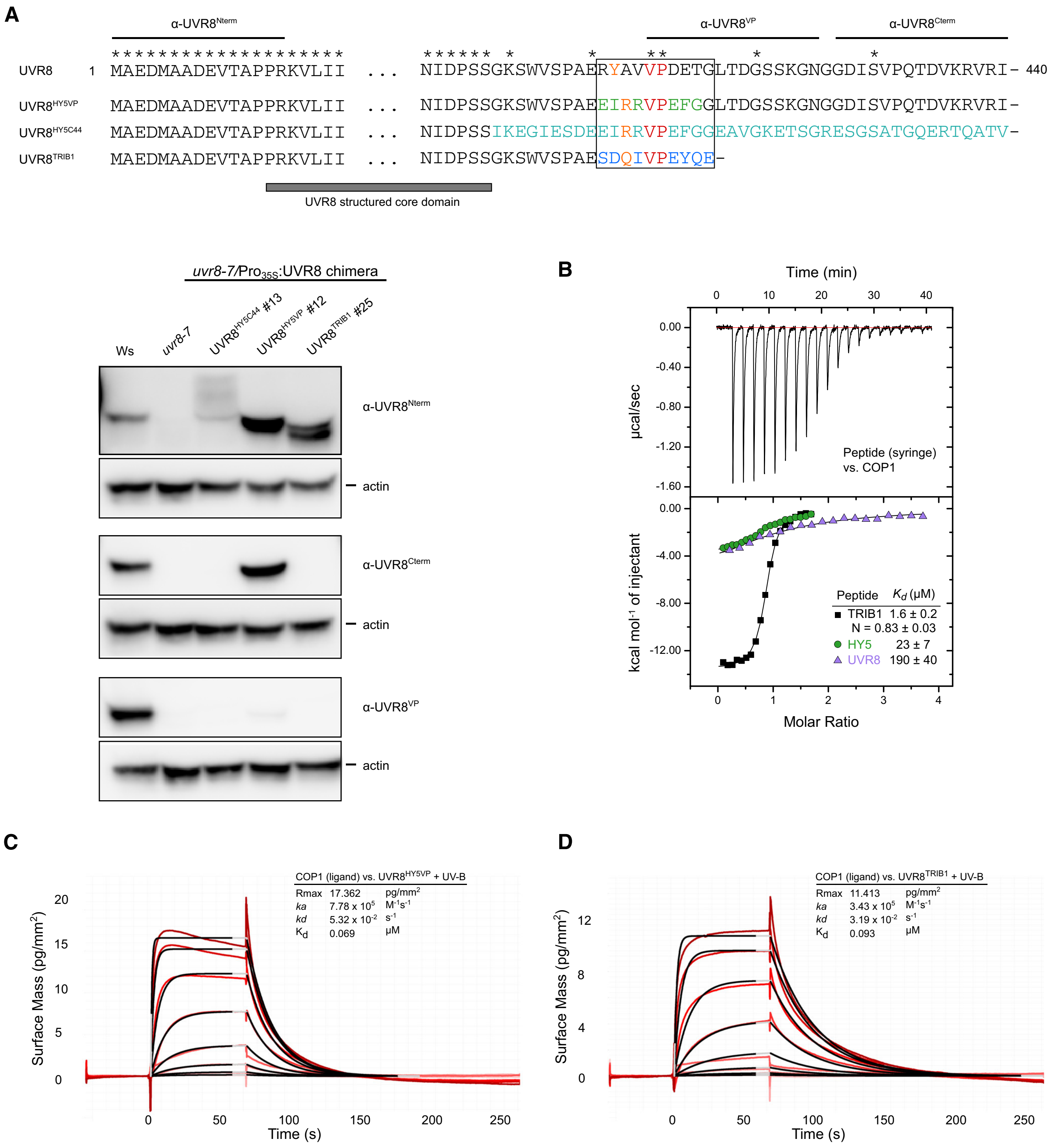
A
α-UVR8Nterm
α-UVR8VP
α-UVR8Cterm
UVR8 1 MAEDMAADEVTAPPRKVLII ... NIDPSSGKSWVSPAERYAVVPDETGLTDGSSKGNGGDISVPQTDVKRVRI- 440
UVR8HY5VP MAEDMAADEVTAPPRKVLII ... NIDPSSGKSWVSPAEEIRRVPEFGGLTDGSSKGNGGDISVPQTDVKRVRI-
UVR8HY5C44 MAEDMAADEVTAPPRKVLII ... NIDPSSIKEGIESDEEIRRVPEFGGEAVGKETSGRESGSATGQERTQATV-
UVR8TRIB1 MAEDMAADEVTAPPRKVLII ... NIDPSSGKSWVSPAESDQIVPEYQE-
UVR8 structured core domain
uvr8-7/Pro35S:UVR8 chimera
Ws
uvr8-7
UVR8HY5C44 #13
UVR8HY5VP #12
UVR8TRIB1 #25
α-UVR8Nterm
actin
α-UVR8Cterm
actin
α-UVR8VP
actin
B
Time (min)
µcal/sec
Peptide (syringe) vs. COP1
kcal mol-1 of injectant
Molar Ratio
Peptide Kd (µM)
TRIB1 1.6 ± 0.2
N = 0.83 ± 0.03
HY5 23 ± 7
UVR8 190 ± 40
C
COP1 (ligand) vs. UVR8HY5VP + UV-B
Rmax 17.362 pg/mm2
ka 7.78 x 105 M-1s-1
kd 5.32 x 10-2 s-1
Kd 0.069 µM
Surface Mass (pg/mm2)
Time (s)
D
COP1 (ligand) vs. UVR8TRIB1 + UV-B
Rmax 11.413 pg/mm2
ka 3.43 x 105 M-1s-1
kd 3.19 x 10-2 s-1
Kd 0.093 µM
Surface Mass (pg/mm2)
Time (s)

Figure EV3.

Figure EV3. UVR8 chimeras can bind the COP1 WD40 domain.

A Immunoblot analysis of UVR8 and actin (loading control) protein levels in 7-day-old wild type (Ws), *uvr8-7* and *uvr8-7*/Pro$_{35S}$:*UVR8^{HY5C44}*, *uvr8-7*/Pro$_{35S}$:*UVR8^{HY5VP}*, and *uvr8-7*/Pro$_{35S}$:*UVR8^{Trib}* seedlings. Specific antibodies against UVR8$^{1–15}$ (α-UVR8^{Nterm}), UVR8$^{410–424}$ (α-UVR8^{VP}), and UVR8$^{426–440}$ (α-UVR8^{Cterm}) were used. Epitopes of the antibodies are indicated on the sequence alignment of the chimeras.

B ITC experiment between the TRIB1 VP peptide versus the COP1 WD40 domain. Integrated heats are shown in solid, black squares. For comparison, ITC experiments between the UVR8 and HY5 VP peptides (from Fig 1B) versus the COP1 WD40 domain are shown in purple triangles and green circles, respectively. The following concentrations were typically used (titrant into cell): TRIB1–COP1 (1,000 μM in 175 μM). The inset shows the dissociation constant (K_d), stoichiometry of binding (N) (± standard deviation).

C, D Binding kinetics of the (C) UVR8^{HY5VP} or (D) UVR8^{TRIB1VP} chimeras pre-monomerized by UV-B versus COP1 obtained by GCI experiments. Sensorgrams of UVR8^{HY5VP} injected are shown in red, with their respective 1:1 binding model fits in black. The following amounts were typically used: ligand—COP1 (2,000 pg/mm^2); analyte—UVR8 chimeras (highest concentration 2 μM). k_a = association rate constant, k_d = dissociation rate constant, K_d = dissociation constant.

Source data are available online for this figure.

Figure EV4. Various VP peptides bind the COP1 WD40 domain.

A–D (left) Crystal structure of the indicated peptide bound to the COP1 WD40 domain. The peptide is depicted in ball-and-stick representation. Selected residues from the COP1 WD40 domain are depicted in gray in stick representation. The TRIB1–COP1 structure is from PDB-ID: 5IGO. (Right) ITC assays between the indicated peptide versus the COP1 WD40 domain or buffer. The following concentrations were typically used (titrant into cell): STO–COP1 (1,500 μM in 150 μM); HYH–COP1 (1,500 μM in 125 μM); and HFR1–COP1 (1,250 μM in 125 μM). See Fig EV3B for the ITC experiment between the TRIB1 peptide versus the COP1 WD40 domain.

 © 2019 The Authors

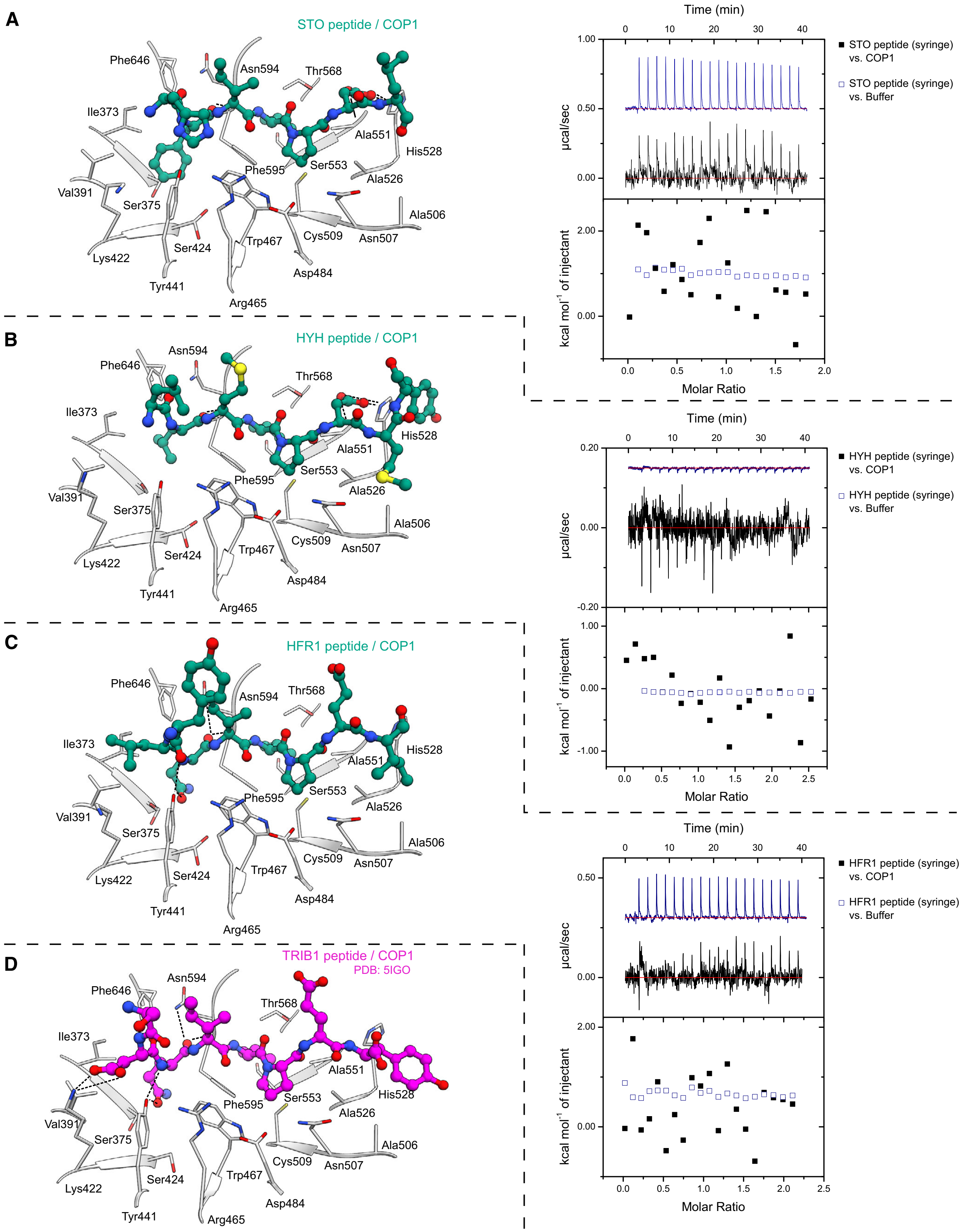
A
STO peptide / COP1
Phe646
Asn594
Thr568
Ile373
Ala551
His528
Phe595
Ser553
Val391
Ser375
Ala526
Ala506
Ser424
Trp467
Cys509
Asn507
Lys422
Asp484
Tyr441
Arg465
B
HYH peptide / COP1
C
HFR1 peptide / COP1
D
TRIB1 peptide / COP1
PDB: 5IGO
Time (min)
µcal/sec
kcal mol-1 of injectant
Molar Ratio
STO peptide (syringe) vs. COP1
STO peptide (syringe) vs. Buffer
HYH peptide (syringe) vs. COP1
HYH peptide (syringe) vs. Buffer
HFR1 peptide (syringe) vs. COP1
HFR1 peptide (syringe) vs. Buffer

Figure EV4.

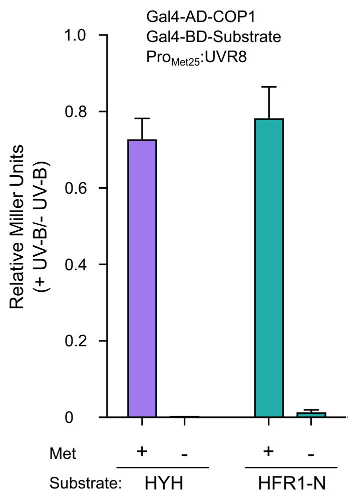

Figure EV5. UVR8 can compete for COP1 binding against other COP1 interactors.

Yeast 3-hybrid analysis of the COP1–HYH and COP1–HFR1 interactions in the presence of UVR8. Normalized Miller Units were calculated as a ratio of β-galactosidase activity in yeast grown under UV-B versus yeast grown without UV-B. Additionally, normalized Miller Units here are reported separately for yeast grown on media without or with 1 mM methionine, corresponding to induction (− Met) or repression (+ Met) of *Met25* promoter-driven UVR8 expression, respectively. Means and SEM for three biological repetitions are shown. AD, activation domain; BD, DNA binding domain; Met, methionine.

 © 2019 The Authors
