# Supplementary material for: Plant photoreceptors and their signaling components compete for COP1 binding via VP peptide motifs
Source: EMBO J. 2019 Jul 15;38(18):e102140. doi: 10.15252/embj.2019102140 (PMC6745501; doi:10.15252/embj.2019102140)

Source Figure EV2A

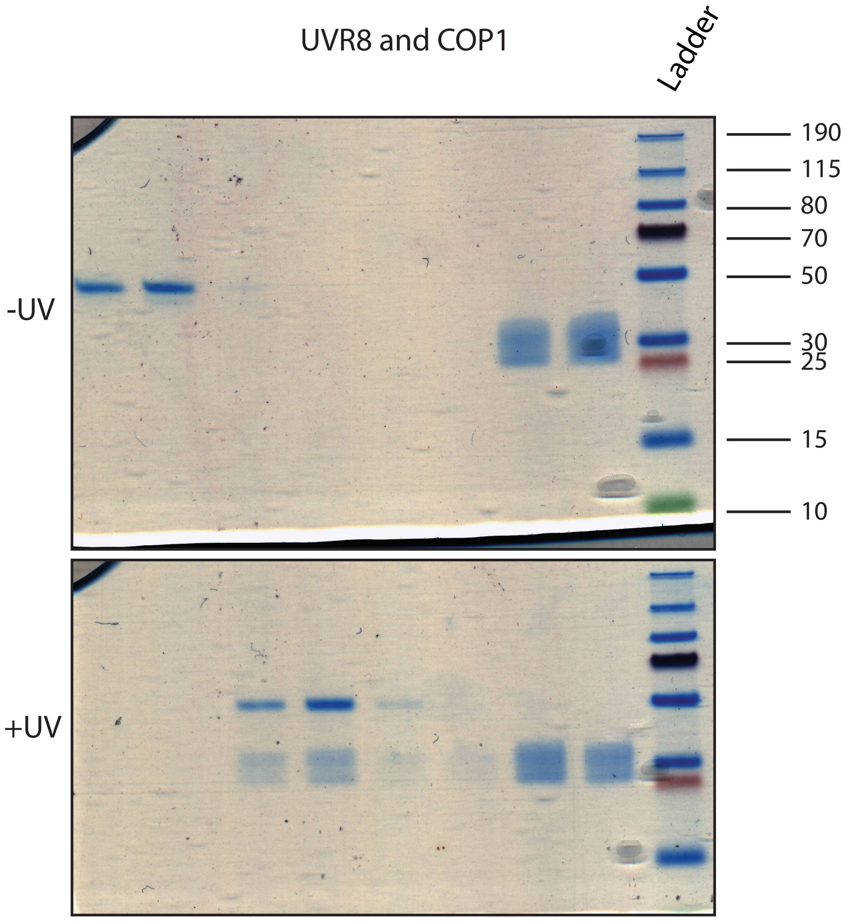

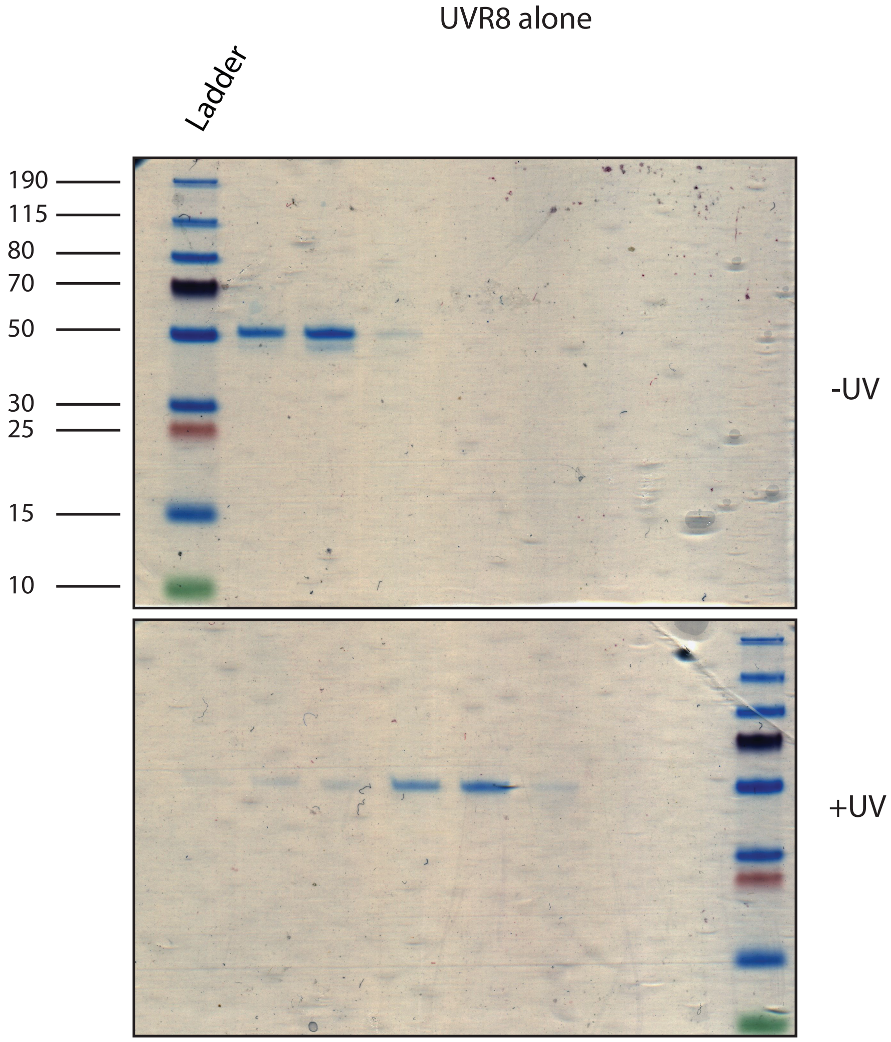

Supplement: Supplementary file 5 — Source Data for Expanded View [file EMBJ-38-e102140-s005.zip › Source_Data_For_EV2A.pdf]
