# Supplementary figures and images for: Plant photoreceptors and their signaling components compete for COP1 binding via VP peptide motifs
Source: EMBO J. 2019 Jul 15;38(18):e102140. doi: 10.15252/embj.2019102140 (PMC6745501; doi:10.15252/embj.2019102140)

# Source Figure EV1A

ECL signal

ECL signal + visible composite

YFP-COP1

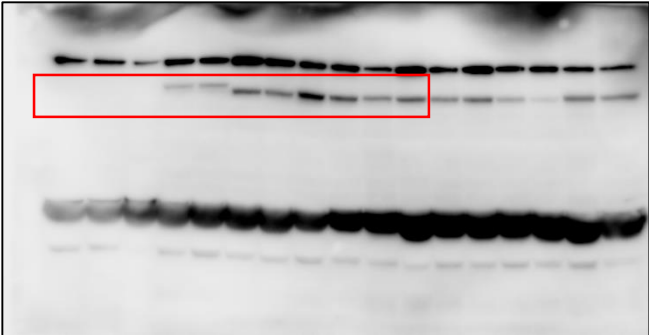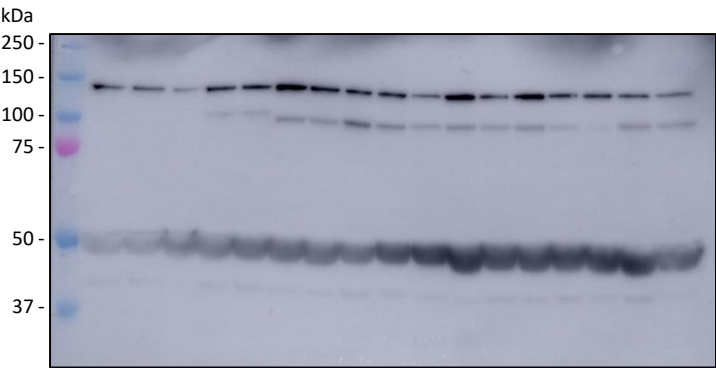

UVR8

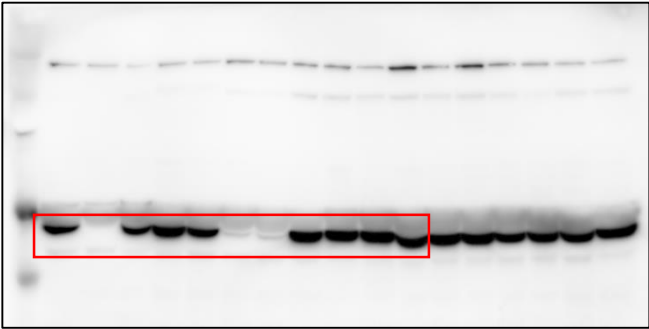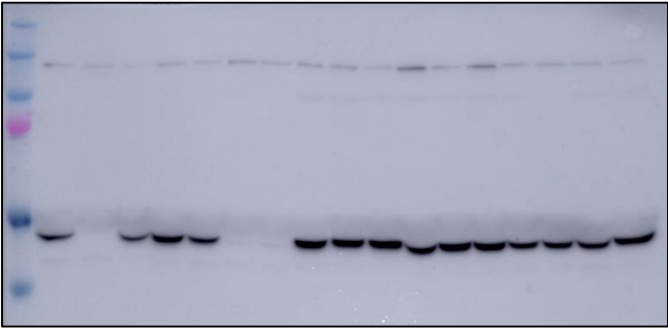

actin

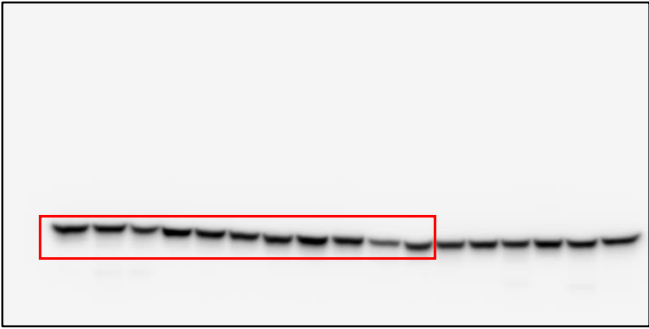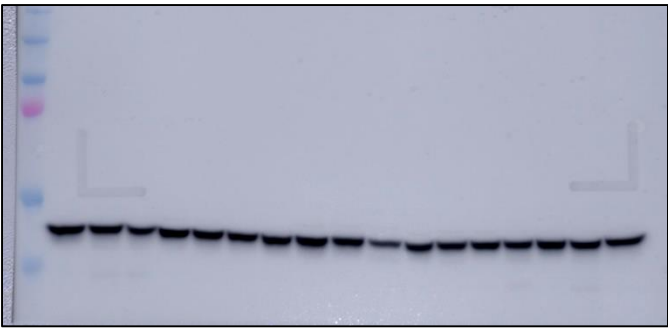

Supplement: Supplementary file 5 — Source Data for Expanded View [file EMBJ-38-e102140-s005.zip › Source_Data_For_EV1.pdf]

Source Figure EV2B

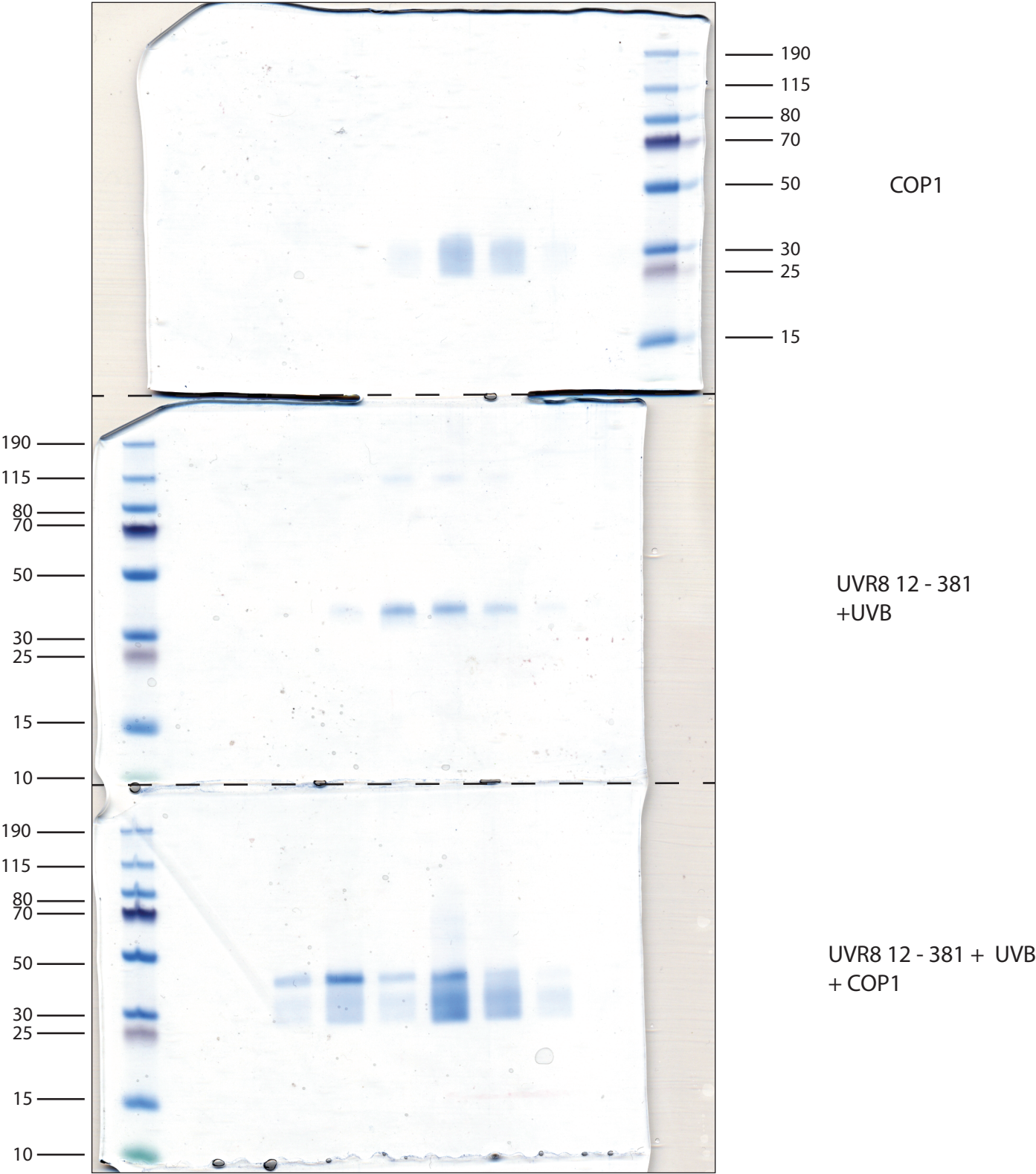

Supplement: Supplementary file 5 — Source Data for Expanded View [file EMBJ-38-e102140-s005.zip › Source_Data_For_EV2B.pdf]

Source Figure EV2C

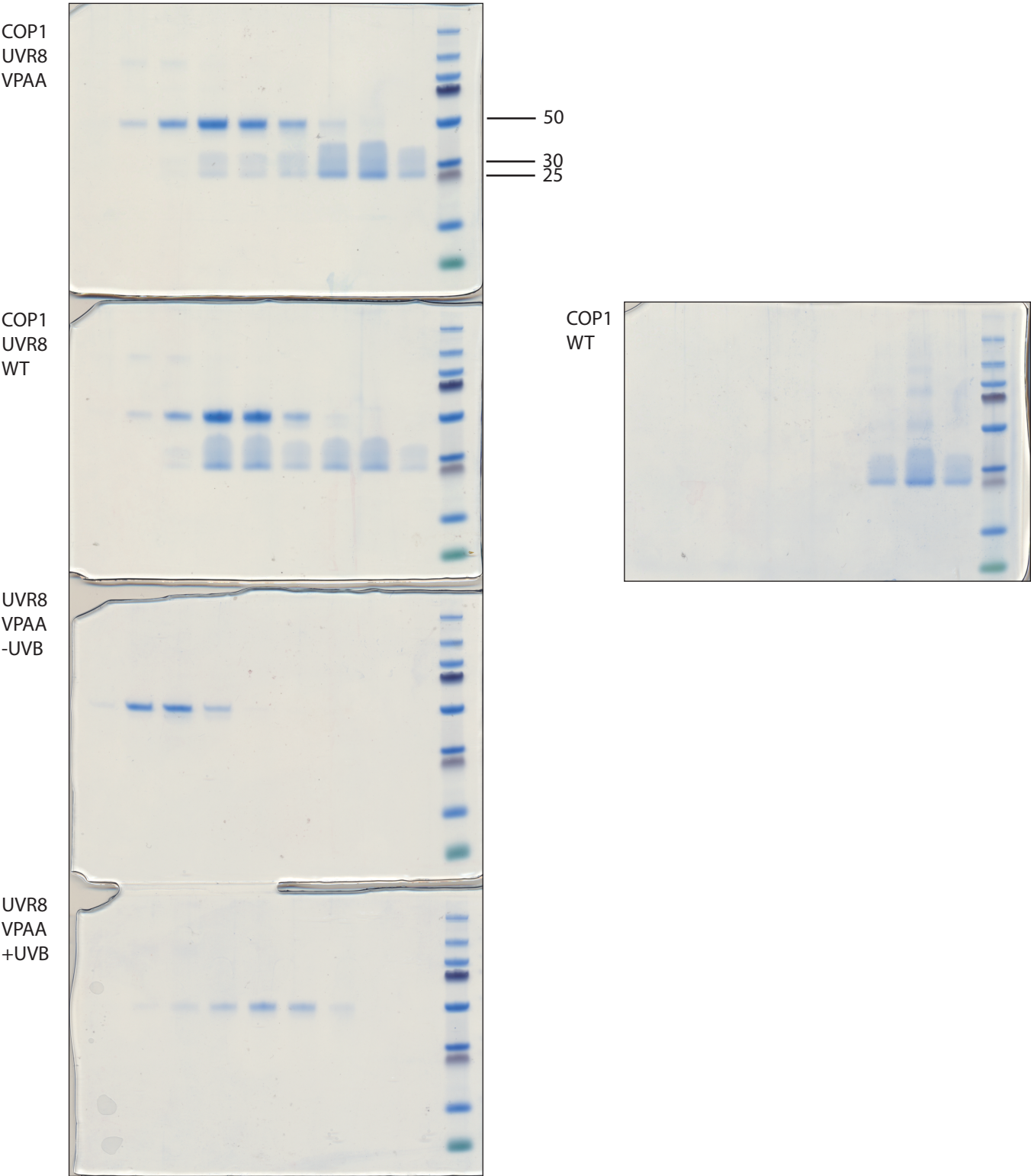

Supplement: Supplementary file 5 — Source Data for Expanded View [file EMBJ-38-e102140-s005.zip › Source_Data_For_EV2C.pdf]

Source Figure 5E

ECL signal

ECL signal + visible composite

CRY2

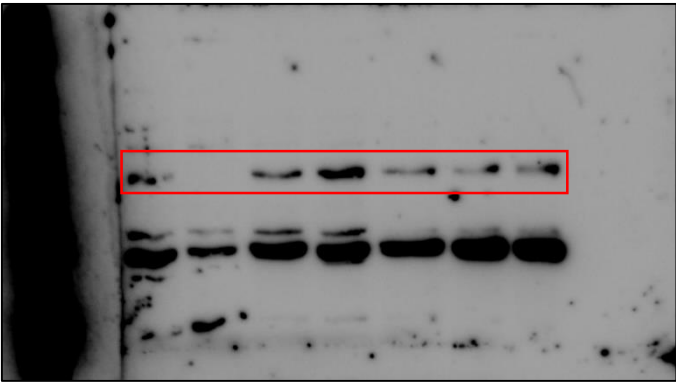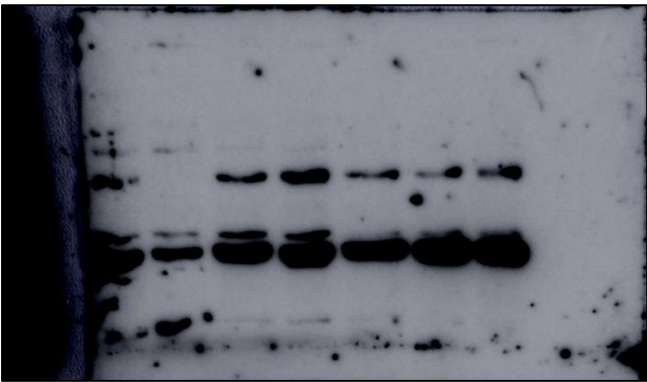

actin

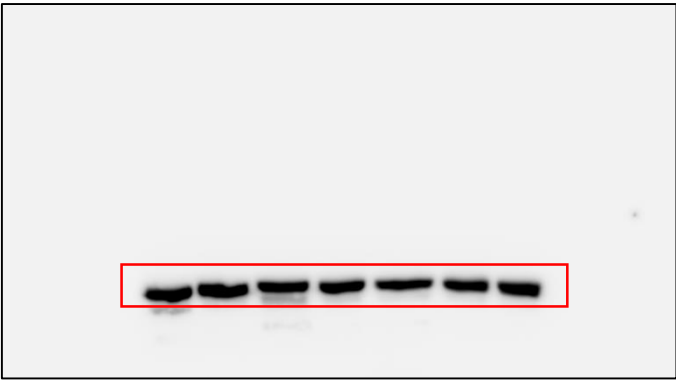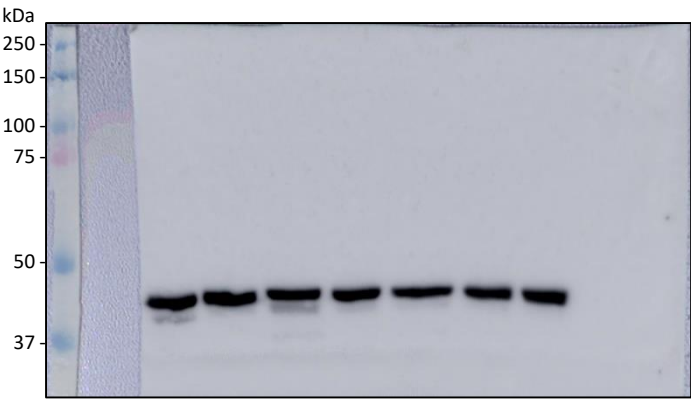

Supplement: Supplementary file 7 — Source Data for Figure 5E [file EMBJ-38-e102140-s006.pdf]
